# Supplementary material for: Spatial Variation in Stream-Water Bacterial Communities Across Sites Differing in Wild Chinese Giant Salamander Detection Frequency
Source: Biology (Basel). 2026 Sep 18;15(18):1647. doi: 10.3390/biology15181647 (PMC13604818; doi:10.3390/biology15181647)
Supplement: Supplementary file 1 [file biology-15-01647-s001.zip › Data Related to “Spatial Variation in Stream-Water Bacterial Communities across Sites Differing in Wild Chinese Giant Salamander Detection Frequency”/Microbial Data/result/02.FeatureAnalysis/02.FeatureAnalysis_ReadMe.pdf]

扩增子结果文件补充说明（02）

2023年8月28日

02.FeatureAnalysis 【特征序列和特征表】

1. feature.fasta 【特征序列】

2. feature.tax_assignments.txt 【物种注释结果】

使用QIIME2 软件进行物种注释。对于16S和18S， 数据库为 Silva 138.1，对于 ITS，数据 库为 Unite v9.0。对于非常规区域，默认用micro_NT数据库注释。

注释结果中出现 mitochondria、chloroplast ，中文翻译为线粒体和叶绿体 ，但是在这里不 要人为的翻译成线粒体和叶绿体 ， 我们注释的数据库里命名都是其官方命名 mitochondria 属于 Proteobacteria（变形菌门 ）， Alphaproteobacteria（α变形菌纲 ）， Rickettsiales（立克次氏体目），是一种原核微生物， chloroplast 在蓝细菌门比较常见的。

3. aligned-rep-seqs.qza 【多序列比对结果】

4. denoisingTable_qza 【去噪丰度qza导出文件】

5. denoisingTable.qza 【去噪丰度qza文件】

6. denoisingTable_qzv 【去噪丰度qzv导出文件】

7. denoisingTable.qzv 【去噪丰度qzv文件】

8. featureSeqs_qza 【代表序列qza导出文件】

9. featureSeqs.qza 【代表序列qza文件】

10. featureSeqs_qzv 【代表序列qzv导出文件】

11. featureSeqs.qzv 【代表序列qzv文件】

12. featureTable_qza 【特征表丰度qza导出文件】

13. featureTable.qza 【特征表丰度qza文件】

14. featureTable_qzv 【特征表丰度qzv导出文件】

15. featureTable.qzv 【特征表丰度qzv文件】

16. masked-aligned-rep-seqs.qza 【过滤去除高变区后的多序列比对结果】

17. rooted-tree_qza 【有根树qza导出文件】

18. rooted-tree.qza 【有根树qza文件】

19. unrooted-tree_qza 【无根树qza导出文件】

20. unrooted-tree.qza 【无根树qza文件】

21. seq_taxonomy_qza 【物种注释qza导出文件】

22. seq_taxonomy.qza 【物种注释qza文件】

23. seq_taxonomy_qzv 【物种注释qzv导出文件】

24. seq_taxonomy.qzv 【物种注释qzv文件】

25. tables【特征表】

25.1 sample/group1/ 【样本及分组的特征表】

25.1.1 featureTable_Absolute 【各（样本，组）绝对丰度特征表】

25.1.1.1 featureTable.*.total.absolute.txt 【各（样本，组）的绝对丰度表】

25.1.1.2 featureTable.*.*.absolute.xls 【各（样本，组）总界门纲目科属种（total,k,p,c,o,f,g, s）水平上的绝对丰度】

列数	列标题	含义
1	Taxonomy	注释结果
2	样本/组名称	各（样本，组）中各物种的绝对丰度值
3	Tax_detail	详细注释信息

25.2 featureTable_Relative 【各（样本，组）相对丰度特征表】

25.2.1 featureTable.*.total.relative.xls 【各（样本，组）均一化处理后的相对丰度】

25.2.2 featureTable.*.*.relative.xls 【各（样本，组）总界门纲目科属(total,k,p,c,o,f,g,s)水平上的相对丰度】

25.3 featureTable_Even【各（样本，组）均一化处理后的绝对丰度特征表

均一化意义及方法： 由于测序深度不一致， 样品之间的序列数有多少， 为了消除实验 误差和人为统计误差， 我们需要把样品的序列数设置在同一深度水平， 尤其针对于样品间的 比较分析需要具有可比性， 所以进行均一化。方法是设置一个阈值(一般是序列数最低的那个样品)，然后从样品中随机抽取阈值所设定的序列条数， 进一步分析。

25.3.1 featureTable..*.*.total.even.txt 【各（样本，组）均一化处理后的绝对丰度】

列数	列标题	含义
1	Taxonomy	注释结果
2	样本/组名称	各（样本，组）中各水平物种均一化后的绝对丰度值
3	Tax_detail	详细注释信息

25.3.2 featureTable.*.*.absolute.xls 【各（样本，组）界门纲目科属种(k,p,c,o,f,g,s)水平上的绝对丰度】

27.4 featureTable_Biom 【各（样本,组）biom文件】

25.4.1 featureTable.*.total.absolute.txt.biom 【 各（样本，组） 绝对丰度biom表】

25.4.2 featureTable.*.total.even.txt.biom 【 各（样本，组）均一化处理后的绝对丰度

biom表】
25.4.3 featureTable.*.total.relative.txt.biom 【各（样本，组）的相对丰度丰度biom表】

25.4.4 featureTable.*.total.absolute.txt.qza 【各（样本，组） 绝对丰度qza格式】
25.4.5 featureTable.*.total.even.txt.qza 【各（样本，组）均一化处理后的绝对丰度qza格式】
25.4.6 featureTable.*.total.relative.txt.qza 【各（样本，组）的相对丰度丰度qza格式】

25.5. featureTable_tableStat【各（样本，组）注释结果统计】

25.5.1 featureTable.briefStatInfo.txt 【各（样本，组）简版注释统计结果】
25.5.2 featureTable.relativeStat.*.xls 【各（样本，组）各层级注释数量统计】
25.5.3 classifiedStat.sample.absolute.{xls,svg,png}【各（样本，组）各层级注释统计结果及可视化展示】

列数	列标题	含义
1	Sample_Name	样本名称
2	Kingdom	注释到界水平上的Tags数
3	Phylum	注释到门水平上的Tags数
4	Class	注释到纲水平上的Tags数
5	Order	注释到目水平上的Tags数
6	Family	注释到科水平上的Tags数
7	Genus	注释到属水平上的Tags数
8	Species	注释到种水平上的Tags数
